# High experienced continuity in breast cancer care is associated with high health related quality of life

**DOI:** 10.1186/s12913-018-2925-0

**Published:** 2018-02-20

**Authors:** Susann Plate, Louise Emilsson, Martin Söderberg, Yvonne Brandberg, Fredrik Wärnberg

**Affiliations:** 1 Department of Surgery, Arvika Hospital, kirurgiska kliniken, Arvika sjukhus, S-670 80 Arvika, Sweden; 2 Primary care research unit, Landstinget Värmland, Sweden; 3 Institute of Health and Society, University of Oslo, Oslo, Norway; 4 Department of Oncology, Växjö Hospital, Växjö, Sweden; 5 Department of Oncology, Pathology, Karolinska Institutet, Stockholm, Sweden; 6 Department of Surgical Sciences, Uppsala University Hospital, Uppsala, Sweden

**Keywords:** Continuity of care, Health-related quality of life, HRQoL, Breast cancer, Patient reported outcome

## Abstract

**Background:**

High experienced continuity is known to be associated with lower needs for supportive care and most likely higher quality of life. On this background, the aim of this study was to investigate if patient-experienced continuity of care was associated with health-related quality of life (HRQoL) in breast cancer patients treated at two different-sized breast cancer units.

**Methods:**

In 2016, two questionnaires, “*Statements on experienced continuity of care”* and *“The European Organisation for Research and Treatment of Cancer Quality of Life Questionnaire Core 30 (EORTC QLQ-C30)”,* were sent out to patients diagnosed between 2011 and 2014 at two different-sized breast cancer units in Sweden. Lead times and other data reflecting medical quality were collected from the patients’ medical records and from the National Swedish Breast Cancer Quality Register.

**Results:**

Of 356 eligible patients, 231 (65%) answered the questionnaires, of whom 218 patients were included in the analyses. A statistically significant association was found between high experienced continuity and high global HRQoL (*p* = 0.03). Continuity was higher at the smaller unit, while no major differences between the units were found regarding medical quality or lead times.

**Conclusion:**

The study found that high experienced continuity and HRQoL was strongly associated. A statistically significant higher continuity of care was found at the smaller unit, in line with what was expected. The absence of clinically relevant differences in lead times and medical quality may indicate that continuity could be achieved without loss of quality.

## Background

Continuity of care is a patient-reported outcome (PROM) defined by Reid et al. as “one patient experiencing care over time as coherent and linked” [1]. Continuity is a matter of how the patient experiences the care coordination and integration over time, regardless of how the healthcare system is organized [2]. Continuity of care has been divided into: *Relational continuity, Informational continuity* and *Management continuity* [1]. *Relational continuity* includes an ongoing therapeutic relationship between a patient and the care providers based on trust, stability and predictability. It comprises past and current care and a plan for the patient’s future [1–3]. *Informational continuity* involves both the way in which information is transmitted between different care providers, and how the information is received and then perceived by the patient, homogeneous, timely and individually [1–4]. *Management continuity* describes how the healthcare system coordinates and adjusts to the patient’s shifting needs over time [1–3]. These three types of patient experienced continuity appear to vary in importance throughout the care pathway and since they are all related and dependent on one another, it is now considered that the total experience of all the three components is fundamental [5, 6].

Breast cancer patients ask for continuity in their cancer care and many claim that they are not receiving this to the extent they expect [7, 8]. Patients have also reported that too little time were offered to them in the consultative visits regarding information and emotional reflections, and that the consultation mostly focused on the physical aspects [9]. Studies have shown that high experienced continuity is associated with lower needs for supportive care and most likely, a higher quality of life [10–13]. As the five-year survival for the breast cancer patient has been improving steadily over the last decades, the number of long term survivors is increasing. Supporting evidence for using PROMs in the development of suitable follow-up models of the care for these patients has been reported [14].

Different definitions of health-related quality of life (HRQoL) exist [15, 16].There is however a broad agreement that HRQoL is the effect of a medical condition and/or its therapy upon a patient, comprising physical function, psychological state, social interaction and somatic sensation [17]. HRQoL is considered to be an important patient-reported outcome reflecting treatment, effectiveness, success and the patient experiences [13, 18].

The aim of this study was to investigate if patient-experienced continuity of care was associated with health-related quality of life in breast cancer patients treated at two different-sized breast cancer units.

## Methods

### Patients

The patients in the study were diagnosed and treated either at the Breast Cancer Unit of Arvika District Hospital (BCU Arvika) or at the Breast Cancer Unit of Uppsala University Hospital (BCU Uppsala) between 2011 and 2014, with all stages of primary breast cancer represented and independent of age. They were identified from the National Swedish Breast Cancer Quality Register. All patients who were treated at BCU Arvika during the chosen period and still alive at the time of the study were invited (*n* = 121) to participate in the study. For each patient from BCU Arvika, two patients were randomly selected from BCU Uppsala and matched by age (+/− 0 years) and time of diagnosis (+/− 3 years) (*n* = 235).

The selected time period implied a follow-up time of one to four years from the date of diagnosis, which we judged suitable for assessment of the experienced continuity. The size of the sample from BCU Arvika determined the size of the total sample.

The two units operate in the same region in Sweden. BCU Uppsala has a catchment area of approximately 350,000 inhabitants while BCU Arvika has a catchment area of approximately 40,000. The units´ treatment routines and care pathways are similar. Neither of the units performs breast reconstruction, instead these patients are referred to the Department of Plastic Surgery. The core of the care providers at BCU Arvika consists of 1–2 breast surgeons and 2 contact nurses. At BCU Uppsala the corresponding numbers are 7 breast surgeons and 2 contact nurses. Accordingly, both units work with contact nurses whose tasks are to coordinate and facilitate the care pathway for the patients. Both units handle the diagnostic work-up, surgery and first policlinic follow-up visit. In addition, BCU Arvika administrates adjuvant treatment such as chemotherapy and endocrine treatments, except radiotherapy. Long-term-follow-up together with palliative care for patients with distant metastases are also provided. At BCU Uppsala all adjuvant treatments are given, and all follow-up are administered by the Department of Oncology and palliative care is managed at another department. Considering the lower number of care providers during the care pathway at BCU Arvika, we expected a higher rating of continuity of care at BCU Arvika than at BCU Uppsala. By comparing lead times, and other variables reflecting medical quality, between the two units we explored if these variables were affected by high continuity.

### Questionnaire collection

In January 2016 an information letter, two questionnaires and a prepaid return envelope were sent out to all patients. After four weeks, a reminder went out to non-responders. If the patient responded to and returned the questionnaires, it was considered as informed consent for inclusion in the study.

### Questionnaires

Two questionnaires were used in the study**. “***Statements on experienced continuity of care”* was used to assess continuity of care. It is a validated questionnaire developed in the United Kingdom (UK) [10, 19]. It is based on qualitative research, using the three elements of continuity (informative, relational and structural) as a theoretical framework [2, 10]. The questionnaire consists of seventeen statements about the patients’ experienced continuity over time, and has been used in other studies of cancer patients in the UK [19, 20]. According to a Cochrane review, this is the only assessment tool known to evaluate continuity over time [7]. The patients respond to the 17 statements of continuity on a five-point Likert scale; 1 = strongly disagree, 2 = disagree, 3 = neutral, 4 = agree and 5 = strongly agree. The minimum score is 17 and the maximum is 85. In the present study, scores of ≥75 were considered as “high” experienced continuity, in conformity with King and co-workers [19].In their study scores of 15 of 17 represented high continuity, which was transformed to our score of 75 out of 85. The questionnaire was translated from English to Swedish and then reversed. The translation was later slightly modified after being tested on five breast cancer patients [21]. Additionally, one question regarding the overall experienced continuity was integrated, inspired by King and co-workers [19]. This to further validate the assessment of experienced continuity: *“Finally, try to estimate your total experience of continuity of care concerning your breast cancer service by placing a mark on the scale 1–10*, *(1 indicating low total experienced continuity and 10 indicating high total experienced continuity)”.*

*The European Organisation for Research and Treatment of Cancer Quality of Life Questionnaire Core 30* (EORTC QLQ-C30) was used to evaluate HRQoL [22]. This is a 30 items questionnaire, comprising five functional scales (physical, role, emotional, social and cognitive functioning), three symptom scales (fatigue, pain, and nausea/vomiting), one scale for global quality of life and six single item scales. Each item is scored from one to four, where 1 = not at all, 2 = a little, 3 = quite a bit and 4 = very much, except for the two items for the global quality of life variable, which ranges from 1 = very poor to 7 = excellent. The concerned time frame for all questions is the past week. The validity and reliability of the Swedish version of the EORTC QLQ-C30 has been evaluated [23, 24].

### Data from medical files and quality registry

Data regarding the patients’ characteristics and variables for medical quality were collected from the patients’ medical files and from the National Swedish Breast Cancer Quality Registry. The variables selected to reflect the medical quality included lead times, methods of diagnosis, whether a pre- and postoperative multidisciplinary therapy conference (MDTC) was conducted, type of surgery, re-operations due to tumour related causes or due to complications and choice of adjuvant therapy. Lead times (LT) were defined as: LT1 = from first contact with the health care to surgery and LT2 = from first contact with the health care to start of adjuvant treatment.

### Statistical methods

The scores from the EORTC QLQ-C30 questionnaire were transformed into a 0 to 100 scale. High scores of the functioning and global quality of life scale represent high levels of HRQoL. Low scores on the symptom scales represent low levels of problems and symptoms [25]. When comparing differences in the EORTC QLQ-C30 scores, a mean difference of 5 or more is considered clinically relevant. A difference of 5 to 9 is considered to be of a “little” clinical importance, 10 to 19 “moderate” and 20 or more of “large” importance [26].

To explore statistically significant differences in the characteristics between groups, Chi-Square test or Fishers’ Exact test for nominal and ordinal data were used. For continuous variables (the continuity scale and the EORTC QLQ-C30), independent sample t-test was conducted. The correlation between HRQoL and experienced continuity was calculated by linear regression adjusted for site and interaction between site and high continuity. *P*-values < 0.05 were considered statistically significant. The SAS version 9.4 was used for all statistical analyses.

## Results

In total, 231 out of 356 patients (65%) responded to the questionnaires; 149/235 (63%) from BCU Uppsala and 82/121 (68%) from BCU Arvika. Thirteen of the 231 patients were excluded; operated at another hospital (*n* = 5), cognitive failure (*n* = 5), and refusal to participate despite having returned the questionnaire (*n* = 3). Thus, a total of 218/356 patients were included constituting the final cohort, 139 patients from BCU Uppsala and 79 from BCU Arvika. The patients’ demographic and clinical characteristics are summarized in Table 1. No statistically significant differences were found between the two BCUs regarding age at diagnosis, mode of detection, number of patients diagnosed 2011–2012 or 2013–2014, tumour size or the proportion of in situ carcinomas. Nor were there any differences in the percentage of node negative cancers or in the proportion of patients with more than 4 positive nodes. The percentage of patients with distant metastases was equal as well. Grade 3 invasive tumours were statistically significant more common at BCU Arvika (*p* = 0.03).

**Table 1:** Patients’ demographics and clinical characteristic, BCU Uppsala and BCU Arvika, 2011–2014

Variables	BCU Uppsala+Arvika	BCU Arvika	BCU Uppsala	*p*-value difference Arvika/Uppsala
	*N* = 218	*N* = 79	*N* = 139	
Age at diagnose (mean)	65.3	65.6	65.1	0.75
Detected by mammography screening	127 (58%)	41 (52%)	86 (62%)	0.15
Diagnosed 2011–2012	81 (37%)	31 (39%)	50 (36%)	0.63
Diagnosed 2013–2014	137 (63%)	48 (61%)	89 (64%)	0.63
Tumour size> 20 mm	147 (67%)	56 (71%)	91 (66%)	0.41
Tumour = non-invasive cancer	18 (8%)	7 (9%)	11 (8%)	0.81
Grade of invasive cancer 1–2-3	57–94-49 (26%–43%–24%)	14–33-25 (19%–46%–35%)	43–61-24 (33%–47%–19%)	0.03
Node negative invasive cancer	141 (65%)	46 (69%)	95 (75%)	0.31
Patients with > 4 positive nodes	10 (5%)	2 (2%)	8 (6%)	0.27
Distant metastasis	7 (3%)	1 (1%)	6 (4%)	0.35

Obtained treatments were decided by the National Guidelines dependent on tumour characteristics. The ratio of patients in the cohort receiving chemotherapy during this period was at BCU Arvika 30%, and at BCU Uppsala 28% (*p* = 0.92). Corresponding numbers for endocrine treatment were 65% at BCU Arvika and 71% at BCU Uppsala(*p* = 0.23) and for radiation therapy 56% at BCU Arvika and 60% at BCU Uppsala (*p* = 0.46). Very few patients had immediate breast reconstruction (2.4% at BCU Arvika and 2.6% at BCU Uppsala).

### Patient experienced continuity

**Fig. 1:**
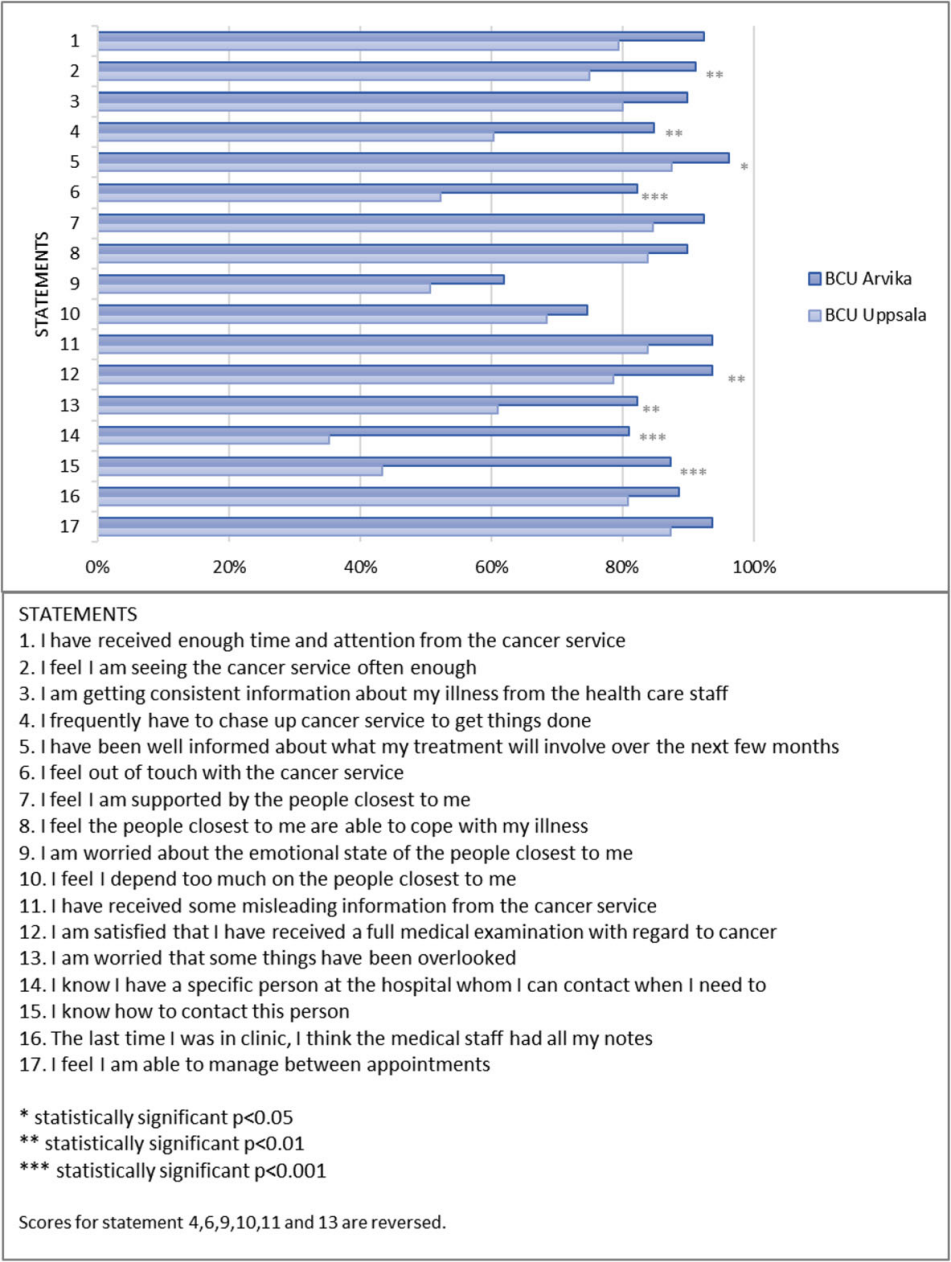
Percent of patients responding “agree” (4) or “strongly agree” (5), indicating an agreement to the 17 statements of continuity, at BCU Arvika and at BCU Uppsala

In the total sample of patients from the two BCUs, the mean score for experienced continuity was 70 (range 33–85) and 37% of the patients scored 75 points or more, the cut off point for high continuity. Mean score for the question regarding total experienced continuity was 8 (range 1–10) (Table 2). The proportion of patients responding agreement (4 or 5 on the Likert scale) to the 17 statements regarding continuity, was lowest (< 60%) for the statement regarding absence of worries for emotional state of the relatives (no: 9) and for the statements regarding the knowledge of whom to contact and how (no: 14 and 15) (Fig. 1). No differences were found regarding time from diagnosis and treatment of breast cancer and the time at which patients completed the questionnaires. (mean difference in score between year individuals diagnosed in 2011–2012 and 2013–2014 was − 0.8 (95%CI =-4,1-2,4,p=0,61 )).

**Table 2:** Experienced continuity at BCU Uppsala and BCU Arvika, 2011–2014

Score	BCU Uppsala+Arvika	BCU Arvika	BCU Uppsala	*p*-value
	*N* = 218	*N* = 79	*N* = 139	
Mean score continuity questionnaire (range 17–85)	70	76	67	< 0.0001
Percent of patients scoring≥75 (= high continuity)	37	61	24	< 0.0001
Mean score “total experienced continuity” (Range 1–10)	8	9	8	< 0.001

Patient-experienced continuity was statistically significant higher in BCU Arvika as compared to BCU Uppsala, (76 and 67 points respectively, *p* < 0.0001). Also, the proportion of patients scoring 75 or more points was higher at BCU Arvika (BCU Arvika 61% and BCU Uppsala 24% *p* < 0.0001) and, the mean score of total experienced continuity was higher at BCU Arvika (BCU Arvika 9 points and BCU Uppsala 8 points, *p* < 0.001) (Table 2). The proportion of patients indicating agreement to the seventeen statements of continuity was statistically significantly higher at BCU Arvika for the statements number 2,4 to 6 and 12 to 15 (Fig. 1). The largest differences between the BCUs were found for the statements 14 and 15, which assess if the patients know whom to contact in the health care organisation and how.

### Health related quality of life (HRQoL)

High experienced continuity (cut-off, ≥75 points) was associated with higher levels in all measured HRQoL scales. The differences in HRQoL between high continuity and low continuity were considered clinically relevant regarding global quality of life, role functioning, emotional functioning, cognitive functioning and fatigue. The differences were also statistically significant for global quality of life (*p* = 0.03), emotional functioning (*p* = 0.006) and fatigue (*p* = 0.02) when adjusting for site (Table 3).

**Table 3:** Association HRQoL/continuity for patients BCU Arvika and BCU Uppsala 2011–2014

Scales in EORTC QLQ-C30	Difference in HRQoL score between patients with high continuity (score ≥ 75) and patients with low continuity (score ≤ 75) (CI)	*p*-value^1^
Global health status/QoL	9 (1–18)^M^	0.03
Role functioning	6 (−5–16)^S^	0.27
Emotional functioning	12(3–20)^M^	0.006
Cognitive functioning	7 (−2–16)^S^	0.11
fatigue^a^	11 (2–21)^M^	0.02
Pain^a^	4 (−8–15)	0.51

CI 95% confidence interval

^1^Calculated by linear regression adjusted for site and interaction in between site and high continuity

^S^Clinical relevance considered to be “Small”, ^M^Clinical relevance considered to be “Moderate”

^a^Low measured score indicates high HRQoL

### Medical quality and lead times

The difference in lead times from the first contact with healthcare to surgery (LT1), BCU Arvika 36 days and BCU Uppsala 40 days was of borderline statistical significance (*p* = 0.05). There was no difference found in lead time from first contact to start of adjuvant treatment (LT2), BCU Arvika 83 days and BCU Uppsala 87 days (*p* = 0.26). No statistically significant differences were found for any of the variables assessing medical quality, with one exception. After BCS, a larger proportion of the patients received postoperative radiotherapy (RT) at BCU Arvika compared to BCU Uppsala. There were no differences between the BCUs regarding patients not receiving treatment in line with the national guidelines (data not shown) and the reason for not following the guidelines was in all cases documented in the medical files (Table 4).

**Table 4:** Medical quality at BCU Arvika and BCU Uppsala, 2011–2014

Variables	Arvika	Uppsala	*p*-value
	*N* = 79	*N* = 139	
MDTC* pre-operative	78	139	0.18
	99%	100%	
MDTC post-operative	100%	100%	_
Type of surgery; BCS** versus mastectomy	49/30	88/51	0.85
	62%/38%	63%/37%	
Postoperative infection within 30 days	7	13	0.85
	9%	10%	
Reoperation done due to surgical complications	1	5	0.41***
	1%	4%	
Reoperation done due to tumour morphology	10	9	0.11
	13%	6%	
Patients with BCS receiving radiotherapy on the breast	43	63	0.01
	92%	73%	

*MDTC Multidisciplinary Therapy Conference, **BCS Breast Conserving Surgery ***Fisher’s Exact Test

## Discussion

In this study, a positive association was found between the breast cancer patients’ experienced continuity of care and HRQoL. Patients who experienced high continuity scored higher regarding HRQoL than those with lower experienced continuity.

The comparison between the two different-sized units showed a statistically significant higher total experienced continuity of care at the smaller unit (BCU Arvika). No differences were found regarding patient characteristics except for a higher occurrence of Grade 3 tumours at BCU Arvika. Regarding medical quality including lead times the only difference was a higher proportion of patient receiving radiotherapy in BCU Arvika than in BCU Uppsala.

The association between high experienced continuity and high HRQoL are in line with earlier studies [10]. These studies, concerned patients with other cancer diagnoses and to our knowledge, no study has solely explored breast cancer patients. It is, however, not possible to decide the direction of these associations. It might be that high HRQoL facilitate high experienced continuity of care, or that high continuity contributes to a higher HRQoL. Future prospective studies are needed to establish if improved continuity will result in better HRQoL.

There is a substantial request for improvement in the continuity of cancer care amongst cancer patients as have been shown in earlier studies [4, 5, 9]. No study has, however, been able to clearly show how to achieve measurable improvements [5, 9]. Implementation of guidelines and pathways, have not been shown to improve the patient’s experienced continuity [5]. The continuity requested by breast cancer patients is further challenged by today’s complex and high specialized care, and the ongoing centralization to larger centers [4].

The relational continuity seems to be central for the patients [7, 27] and, it might not be possible to replace the patient’s request for a relationship with the person who owns the medical responsibility [5]. In our study, an organization of care based on contact nurses, could not bridge all the side effects on continuity that large size and multiple professionals imply. At the larger BCU, the patients’ contact nurse also changed over time as the nurses were either working at the Department of Surgery or at the Department of Oncology. In accordance, the largest difference between the BCUs were concerning knowledge about whom to contact and how. There seems to be room for improvement of these aspects of continuity.

The occurrence of high grade invasive tumours, Grade 3, was statistically significant higher at BCU Arvika (*p* = 0, 03). For that reason, we did a comparison between the proportion of Grade 3 tumours in the two counties based on data from the National Swedish Breast Cancer Quality Register 2007 to 2012 (*n* = 2826) and no difference was found.

There were no big differences regarding medical quality and lead times between the two BCUs. We expected a higher experienced continuity at the smaller BCU and we looked at medical quality and lead time in order to evaluate if continuity could be achieved without a loss of quality, as has been considered [5]. The only statistically significant difference between the BCUs was regarding RT. At BCU Uppsala, many low risk women did not receive radiotherapy as a part of a clinical study, which could explain this difference. The study was later open for BCU Arvika with the possibility to include patients.

The cross-sectional design, asking the patients about previous perceptions of continuity can be regarded as a limitation of the study. However, we considered it to be vital that the patient had experienced the care pathway from diagnosis to follow-up, to be able to have an opinion about the continuity of care. To make it possible for the patient to recall their care trajectory, the time from diagnosis was maximised to four years. A shorter follow-up time would, on the other hand, have make it difficult to experience high or low continuity. HRQoL was however assessed within the last week from questionnaire completion.

The response rate was 65%, which should be considered when interpreting the results. The non-responders were mainly found among the older patients (> 80 years) and among the youngest (< 35 years). Other characteristics of the non-responders were not possible to explore since responding to the questionnaire was considered as the informed consent for reviewing the medical files.

No available generally accepted assessment tool for continuity of care were found at the time of planning the study. The questionnaire selected for this study, has not previously been used in Sweden, although tested in cancer care in the UK [19, 20]. It was formally translated. We found the Swedish version easy to use, and the feasibility for Swedish breast cancer patients was tested with one additional question regarding the total experienced continuity. The score from this additional question was highly associated with the total score from the 17-itemquestionnaire, thus the questionnaire appears to have content validity.

The strengths of the study include that the continuity questionnaire measured the total experience of all the three components of continuity of care from diagnosis to follow-up [7, 19]. In addition, the assessment of HRQoL was performed with a Swedish version of EORTC QLQ-C30, a questionnaire evaluated for validity and reliability and already used in several studies [23, 24]. All data regarding lead times were collected from the patients’ medical records. Medical quality variables were collected from the National Swedish Breast Cancer Quality Registry but, checked in the medical records if missing. The data sources are considered to be of high quality.

## Conclusions

In conclusion, high experienced continuity and HRQoL were strongly associated. There was a higher continuity of care at the smaller unit with minor differences regarding medical quality and lead times. This may indicate that high continuity could be achieved without a loss of quality. To enable best possible HRQoL for the individual patient, prospective studies of interventions using standardised assessment tools are required and encouraged.

## Abbreviations

BCU: Breast Cancer Unit
EORTC QLQ-C30: The European Organisation for Research and Treatment of Cancer Quality of Life Questionnaire Core 30
HRQoL: Health-related Quality of Life
LT: Lead Time
MDTC: Multidisciplinary Therapy Conference
RT: Radiotherapy. BCS=Breast Conserving Surgery

## References

[1] Reid RH Haggerty J, McKendry R. Defusing the confusion: concepts and measures of continuity of healthcare. Ottawa: Canadian Health Services Research Foundation; 2002. http://www.hpm.org/Downloads/Bellagio/Articles/Continuity/cr_contcare_e.pdf.

[2] Haggerty JL, Reid RJ, Freeman GK, Starfield BH, Adair CE, McKendry R (2003). Continuity of care: a multidisciplinary review. BMJ (Clinical research ed).

[3] Waibel S, Henao D, Aller MB, Vargas I, Vazquez ML (2012). What do we know about patients’ perceptions of continuity of care? A meta-synthesis of qualitative studies. International journal for quality in health care : journal of the International Society for Quality in Health Care.

[4] Lafferty J, Rankin F, Duffy C, Kearney P, Doherty E, McMenamin M, Coates V (2011). Continuity of care for women with breast cancer: a survey of the views and experiences of patients, carers and health care professionals. European journal of oncology nursing. the official journal of European Oncology Nursing Society.

[5] Guthrie B, Saultz JW, Freeman GK, Haggerty JL (2008). Continuity of care matters. BMJ (Clinical research ed).

[6] Masood S (2008). Survivorship: a needed program for the continuity of care for breast cancer patients. Breast J.

[7] Aubin M, Giguere A, Martin M, et al. Interventions to improve continuity of care in the follow-up of patients with cancer. Cochrane Database of Systematic Reviews. 2012;11(7). Art. No.: CD007672. 10.1002/14651858. 10.1002/14651858.CD007672.pub2 PMC11608820 22786508

[8] Pennery E, Mallet J (2000). A preliminary study of patients’ perceptions of routine follow-up after treatment for breast cancer. European journal of oncology nursing : the official journal of European Oncology Nursing Society.

[9] McCaughan E, McSorley O (2007). Consumers’ and professionals’ perceptions of a breast cancer review clinic. J Adv Nurs.

[10] King M, Jones L, Richardson A, Murad S, Irving A, Aslett H, Ramsay A, Coelho H, Andreou P, Tookman A (2008). The relationship between patients’ experiences of continuity of cancer care and health outcomes: a mixed methods study. Br J Cancer.

[11] Van Walraven C, Oake N, Jennings A, Forster AJ (2010). The association between continuity of care and outcomes: a systematic and critical review. J Eval Clin Pract.

[12] Tsianakas V, Robert G, Maben J, Richardson A, Dale C, Wiseman T (2012). Implementing patient-centred cancer care: using experience-based co-design to improve patient experience in breast and lung cancer services. Support Care Cancer.

[13] Manary MP, Boulding W, Staelin R, Glickman SW (2013). The patient experience and health outcomes. N Engl J Med.

[14] Warrington L, Absolom K, Velikova G (2015). Integrated care pathways for cancer survivors – a role for patient-reported outcome measures and health informatics. Acta Oncol.

[15] Guyatt GH, Feeny DH, Patrick DL (1993). Measuring health-related quality of life. Ann Intern Med.

[16] Ahmed S, Berzon RA, Revicki DA, Lenderking WR, Moinpour CM, Basch E, Reeve BB, Wu AW (2012). The use of patient-reported outcomes (PRO) within comparative effectiveness research: implications for clinical practice and health care policy. Med Care.

[17] Cella DF (1995). Measuring quality of life in palliative care. Semin Oncol.

[18] Schipper H, Clinch JJ, CLM O (1996). Quality of life and Pharmacoeconomics in clinical trials.

[19] King M, Jones L, McCarthy O, Rogers M, Richardson A, Williams R, Tookman A, Nazareth I (2009). Development and pilot evaluation of a complex intervention to improve experienced continuity of care in patients with cancer. Br J Cancer.

[20] Jones L, Fitzgerald G, Leurent B, Round J, Eades J, Davis S, Gishen F, Holman A, Hopkins K, Tookman A (2013). Rehabilitation in advanced, progressive, recurrent cancer: a randomized controlled trial. J Pain Symptom Manag.

[21] Harkness JA, Villar A, Edwards B et al. Wiley series in survey methodology. Surveymethods in multinational, multiregional, and multicultural contexts. Hoboken: Wiley; 2010. https://dx.doi.org/10.1002/9780470609927.

[22] Aaronson NK, Ahmedzai S, Bergman B, Bullinger M, Cull A, Duez NJ, Filiberti A, Flechtner H, Fleishman SB, de Haes JC (1993). The European Organization for Research and Treatment of cancer QLQ-C30: a quality-of-life instrument for use in international clinical trials in oncology. J Natl Cancer Inst.

[23] Bergman B, Sullivan M, Sorenson S (1992). Quality of life during chemotherapy for small cell lung cancer. II. A longitudinal study of the EORTC Core quality of life questionnaire and comparison with the sickness impact profile. Acta oncologica (Stockholm, Sweden).

[24] Derogar M, van der Schaaf M, Lagergren P (2012). Reference values for the EORTC QLQ-C30 quality of life questionnaire in a random sample of the Swedish population. Acta Oncol.

[25] Fayers P, Aaronson NK, Bjordal K, Groenvold M, Curran D, Bottomley A. EORTC. QLQ-C30 scoring manual, 3rd ed. Brussels: European Organisation for Research and Treatment of Cancer. 2001. http://www.eortc.be/qol/files/SCManualQLQ-C30.pdf. Accessed May 2016.

[26] Osoba D, Rodrigues G, Myles J, Zee B, Pater J (1998). Interpreting the significance of changes in health-related quality-of-life scores. Journal of clinical oncology : official journal of the American Society of Clinical Oncology.

[27] Wagner EH, Aiello Bowles EJ, Greene SM, Tuzzio L, Wiese CJ, Kirlin B, Clauser SB (2010). The quality of cancer patient experience: perspectives of patients, family members, providers and experts. Quality & safety in health care.

